# PROTOCOL: Interventions to improve the economic self‐sufficiency of unemployed immigrants from non‐Western countries

**DOI:** 10.1002/cl2.1115

**Published:** 2020-09-28

**Authors:** Frederik Thuesen, Vibeke Jakobsen, Nina T. Dalgaard, Bjørn C. A. Viinholt

**Affiliations:** ^1^ VIVE—The Danish Center for Social Science Research Copenhagen Denmark

## Abstract

**Background:**

This is a protocol for a Campbell Review. The objectives are as follows:

**Objectives:**

This review systematically collects and synthesizes evidence from evaluations of causal effects of interventions designed to improve employment outcomes for non‐Western immigrants. The review aims to answer the following questions:

1) Do interventions designed to improve the economic self‐sufficiency for non‐Western immigrants affect participants employment, use of cash assistance, income, or job retention?
2) Do effects differ depending on programme content or populations served?

## 1 BACKGROUND

### 1.1 Description of the condition

In 2015 permanent migration to the OECD countries reached its' highest level since 2007 with 4.7 million entries—partially due to the surge in refugees during recent years (OECD, 2017a). Immigrants can be refugees, displaced persons, economic migrants and persons moving for other purposes, including family reunification (Dumont, Thomas, Jorg, Filip, & Theodora, 2016, p.4). Although in the OECD countries more than two in three immigrants are employed, unemployment affects immigrants, and especially refugees, to a larger extent than the rest of the population. The average unemployment rate of immigrants was 8.3% in 2016 in all OECD countries and 12.4% in the European OECD countries. This is, respectively, 1.8 and 4.3 percentage points higher than the rate of native workers. However, in some OECD countries the gap is much larger. In countries such as Belgium, France, Spain and Sweden the employment gap between native and foreign‐born workers is between 7.5% to 11.0% in 2016. In other OECD countries, such as the Slovak Republic and Israel, foreign‐born workers are in fact employed to a higher extent than native workers.^1^

These unemployment figures cover all immigrants in the OECD countries. However, there are large differences between immigrant groups with respect to labour market integration. In 2018, the unemployment rate in the EU‐28 was: 6.1% for the native born population, 6.8% for persons born elsewhere in the EU and 12.2% for persons born outside the EU respectively (Eurostat, 2019). For the United States, Borjas have shown that immigrants from Europe, Canada and Australia are in general more successful in the American labour market than immigrants from Africa and Asia (Borjas, 2004).^2^ Possible explanations for these differences in labour market integration by country of origin include differences in the composition of immigrants with respect to skill level and residence type, differences in language and cultural distance between the country of origin and the country of destination, and differences in the level of labour market discrimination of different immigrant groups (Chiswick and Miller, 2001; Dumont et al., 2016; Fleischman & Dronkers, 2010; Schultz‐Nielsen 2019; Tubergen, Maas, & Henk, 2004).

Unemployment is a challenge to economic self‐sufficiency and the well‐being of the affected immigrants including refugees (Kennedy & Ted, 2006; Lindert, Ehrenstein Ondine, Stefan, Andreas, & Elmar, 2009; Roelfs, Shor, Davidson Karina, & Schwartz Joseph, 2011). This is due to the fact that the immigrant unemployment may give rise to mental health problems, social exclusion, poverty and deprivation (e.g., ability to afford rent and nutritious foods; Andersen, Jorsal, Jørgensen, Koob, & Thomsen, 2018; Strandh, Anthony, Karina, & Anne, 2014). Moreover, the relatively low employment rates of immigrants affect public finances in destination countries with comprehensive social protection systems negatively, due to lower average tax contributions from unemployed immigrants (OECD, 2013). Therefore, labour market integration of immigrants in the form of economic self‐sufficiency is a central political goal to most destination countries.

Different countries deploy different programmes to raise the employment level of immigrants. Some of these interventions are specific to unemployed immigrants—such as introduction programmes for language training to recently arrived refugees. Other programmes are not specific to immigrants but deployed to further re‐employment prospects among unemployed citizens in general such as active labour markets programmes (ALMPs), for example, coaching or mentoring, on‐the‐job‐training or subsidised employment. A gap in the literature remains in terms of outcomes from interventions aiming at improving immigrant (including refugee) economic self‐sufficiency. This review will look at research on the outcome of programme participation (i.e., effects *during* and *after* programme participation) for non‐Western immigrants. Western countries are defined as EU28/EEA plus the United States, Canada, Australia and New Zeeland. Non‐Western countries are all other countries. We want to focus on interventions seeking to assist the least successful groups of immigrants (with respect to the labour market). Thus we focus on non‐Western immigrants. We are aware that the terms “non‐Western/Western” are not perfect and may be seen as ethnocentric. However, we are not aware of any different terminology, which would be more suitable. Therefore, to our knowledge this categorisation is the best we dispose of in order provide readers with succinct analytic terms.

### 1.2 Description of the intervention

The review will define as eligible any intervention designed to increase the economic self‐sufficiency and reduce unemployment rates of immigrants (i.e., refugees, displaced persons, economic migrants and persons moving for other purposes, including family reunification). The review adopts a relatively broad perspective on interventions targeting immigrants' economic self‐sufficiency given the sparse knowledge on the effectiveness of interventions and the limited number of previous systematic reviews in this field. With a few modifications we will tentatively classify these interventions along the lines proposed by Butschek and Walter (2014). Butscheck and Walter distinguish between two different types of programmes: first, migrant specific programmes, that is, programmes specifically designed for and exclusively targeted at immigrants; second, general ALMPs, that is, general programmes also used for the native population. To these two types of programmes we will add a third type that encompasses combinations of different types of programmes, that is, combination programmes. If we find studies on the effect of interventions that cannot be placed into one of the described categories or if there is too much overlap between some of the categories, we will adjust the categorisation.

General ALMPs comprise four types of interventions—following classifications from OECD (OECD, 2004) and Eurostat (Eurostat 2005)^3^:

1. **(Labour market) training** are programmes that can be classroom training, on‐the‐job training or work experience. Training may also provide general education (language courses, basic computer courses) or specific adult vocational training and certificates. It may also provide specific vocational skills (advanced computer courses or courses providing technical or manufacturing skills). The basic purpose of training is to develop the productivity and employability of the citizen through enhancing human capital. Training is a classical type of ALMP. Previous studies have shown that it is important to distinguish between classroom‐training and on‐the‐job training (Arendt & Pozzoli, 2013, Arendt, Pohl Nielsen, & Jakobsen, 2016). Therefore, we will divide the training programmes into two subgroups: (1a) classroom training and (1b) on‐the‐training/work experience.
2. **Subsidised private sector employment** are programmes that create incentives to alter employer and/or worker behaviour in relation to private sector employment. Typically, these programmes encompass temporary wage subsidies to the employer that aim at encouraging him or her to hire workers or maintain jobs that might otherwise be broken up. Private sector programmesmay also encompass financial incentives toworkers, for example, in the formof either reduced or full salary for the hours worked. Such programmes may also encompass self‐employment grants to start up a business along with advisory support on how to do this.
3. **Subsidised public sector employment** are programmes that aim at direct creation and provision of public works or other activities that produce public goods or services. Often these programmes are targeted at the most disadvantaged individuals who are at risk of long‐term unemployment or falling out of the labour market. Hence these programmes aim at continuing contact with the labour market and its requirements and at preventing loss of human capital.
4. **Job search assistance** are programmes that aim at enhancing job search efficiency. The programmes may include job search courses, CV counselling, job clubs, vocational guidance, counselling and monitoring of job search efforts. Public employment services (PES) or private agencies may provide such assistance. Often public employment administration (state‐led or municipal) delivers assistance for the most disadvantaged job‐seekers while private agencies or private unemployment insurance funds provide assistance for the more privileged employees, for example, white collar workers.
  We should add a fifth type of labour market intervention that is not in itself an ALMP but a typical additional element to ALMPs and some migrant‐specific programmes such as language training:
5. **Sanctions and economic incentives**. In many countries, legislation requires sanctions for unemployment benefit‐recipients who fail to live up to integration programmes or unemployment legislation and rules. The OECD notes in relation to language courses targeting immigrants that “countries are increasingly turning towards incentivising the acquisition of language skills” by linking completed language tests either to different types of economic rewards or to different permit decisions (OECD, 2018a, p. 101). Moreover, public authorities may impose benefit sanctions, for example, short or long‐term reduction or suspension of unemployment benefits, on job seekers, natives or immigrants, who fail to provide sufficient job search activity or refuse an acceptable job offer. To the extent that sanctions target benefits, they function as an economic incentive. Economic incentives may also be part of a general integration policy encompassing, for example, reduced income transfers to unemployed immigrants (Rosholm & Rune, 2010).
  Migrant‐specific programmes encompass two categories:
6. **Language training**: Many countries provide language training to immigrants—either as part of an introduction programme or as a separate programme (OECD 2018a, pp. 100–101). Language skills are typically regarded as a crucial determinant of immigrants employment outcomes and earnings as well as successful social and workplace integration. Research shows that the economic returns to language proficiency in general are large. Comparing immigrants with strong and weak destination country language proficiency, different studies have found substantially lower employment and income among members of the latter group (Aldashev, Gernandt, & Thomsen, 2009; Dustmann & Fabbri, 2003). Therefore, such programmes potentially have sizeable effects. Immigrants may acquire destination country language skills through informal activities (e.g., self‐study or learning by doing), but formal training may accelerate the process of language acquisition. Language programmes may include training teachers in second language acquisition. Often these programmes include teaching components focusing on history, culture and institutions of the destination country. The German so‐called “Living in Germany Orientation Course” is one example of such a course (Liebig, 2007). However, language programmes may also target teaching specific occupational vocabularies if deemed appropriate.
7. **Introduction programmes** are programmes that aim at facilitating transition from immigration to labour market and social integration. Introduction programmes are primarily a combination of language courses, general orientation on the destination country culture and institutions as well as labour market programmes (Joona & Lena, 2012). For newly arrived immigrants they typically start with language training and continue with other training, job internships or subsidised employment. In some countries such as the Nordic countries, these programmes encompass a customised integration plan toward employment uptake. In Sweden, introduction programmes have been offered since the 1960s (Andersson & Nekby, 2012), in Denmark since the 1990s (Clausen, Hummelgaard, Leif, Blume, & Michael, 2006).
  To the seven programmes mentionned above, we want to add an eighth category:
8. **Combination programmes**: A recent article by Card et al. (2018) add to the four ALMP types mentioned above a category they term “Other programmes combining two or more of the above types”. They add that most of such programmes combine an element of job search with training or subsidised employment. Hence, we also find it relevant to include among the reviewed interventions a programme type that captures combinations of different ALMPs (i.e., category type 1–5) as well as language‐training (category 6). Such programmes may also include what Butchek and Walter (2014) term general programmes exclusively for immigrants that consist in general ALMPs other than language courses. Often, these programmes combine different ALMPs (e.g., job search, training and sanctions). Aslund and Johansson (2011) describes such a programme type in Sweden. The programme consists in intensified job search assistance that assigns immigrants to caseworkers whose caseload has been reduced. However, we find the exclusion of language training from such a combination category suboptimal given that language training can be part of an introduction programme but also a separate programme that can be combined with other programmes. We exclude introduction programmes (i.e., category 7) from our combination programme category since introduction programmes are also, typically, combination programmes. Therefore, in order to distinguish combination programmes targeting newly arrived immigrants from other combination programmes we find it most suitable to keep those two categories separate.

Eligible interventions may be assigned by public, that is, state, regional or municipal, authorities, or by private for‐profit or nonprofit actors on behalf of public authorities (although sanctions and economic incentives are typically administered by public authorities). Some of these programmes demand full‐time participation for long periods (e.g., months or, in the case of introduction programmes, years) while other programmes have a shorter duration (e.g., a few days or weeks).

These interventions will be compared to a control or comparison group receiving no interventions (passive benefits) or “services as usual” or alternative interventions. The review will not include interventions fully financed and implemented by civil society organisations (NGOs) since the purpose of this review is to evaluate the effects of programmes designed as elements in a public labour market integration policy.

### 1.3 How the intervention might work

**Figure 1:**
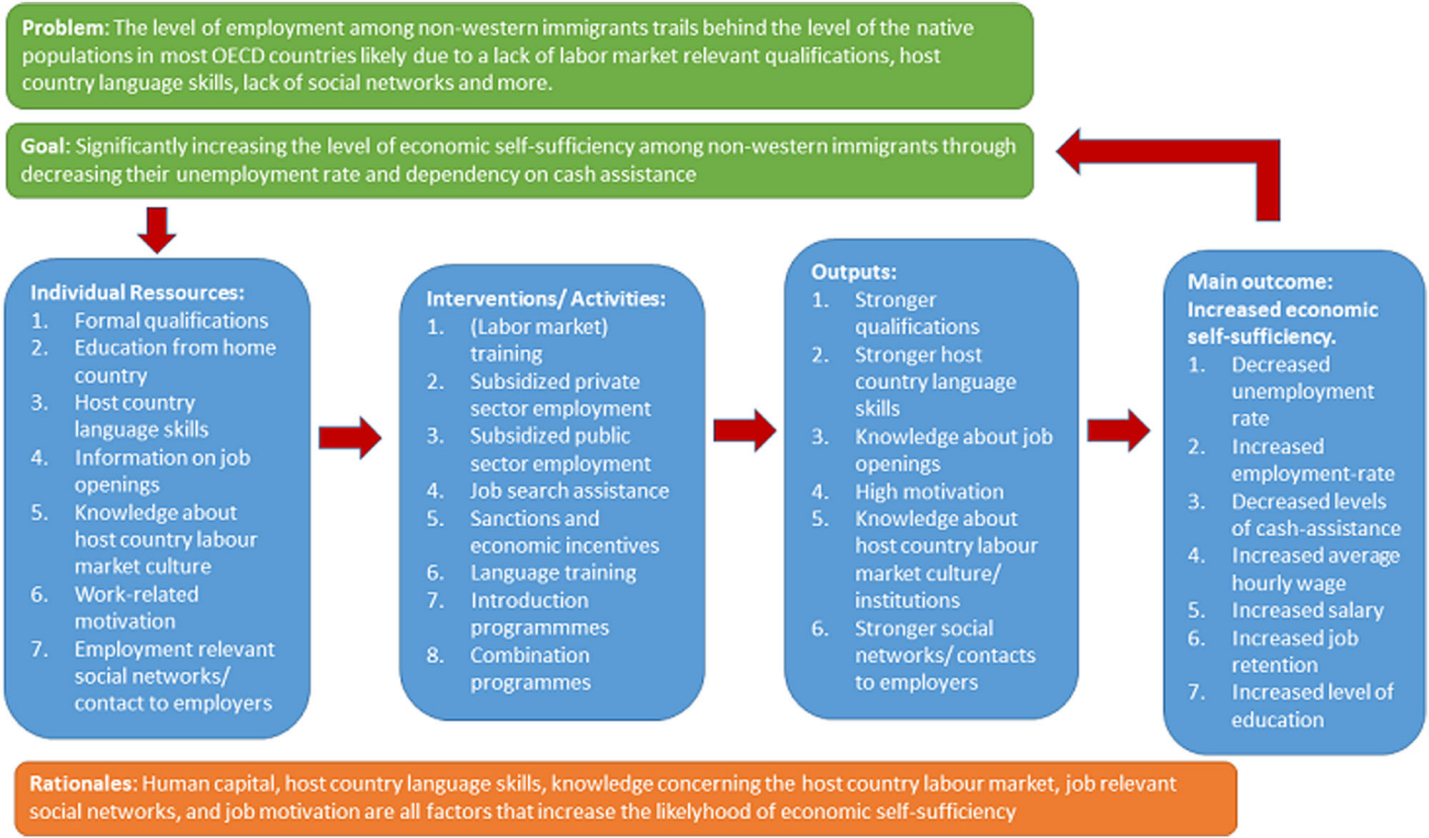
Logic model connecting labour market programmes to effects on immigrant's economic self‐sufficiency

The programmes described in the previous section seek to provide the individual immigrant with competencies and resources enhancing the likelihood of labour market integration, including competencies important for successful enrolment and completion of a formal education that increases individual chances of finding employment on the medium/longer term. Through different types of courses, training, counselling, and incentives these programmes seek to enhance resources such as human capital (qualifications and labour market experience), destination country language competencies, knowledge about the local labour market and its work culture, access to job relevant networks (social capital), and a work ethic or motivation compatible with the standards of the local labour market. Immigrant labour market integration research accounts for the mechanisms that these programmes seek to activate. In immigrant labour market integration research, the human capital model is the dominant paradigm (Kogan, 2011; Kogan, Frank, Elisabeth, & Yinon, 2011). Research shows that immigrants with higher levels of human capital‐primarily in terms of education and labour market experience are more likely to integrate quickly and successfully into the destination country society (Chiswick, 2005; however see also Arendt, Pohl Nielsen, et al., 2016). Such success is more likely the more such education and labour market experience is similar to the educational credentials and the experience that can be obtained in the destination country (Ebner & Helbling, 2016). Research has also shown that good destination country language skills matter positively to the employment chances of immigrants (Chiswick & Miller Paul, 2003; Isphording, 2015; Kossoudji, 1988). Moreover, good information on job openings and specific knowledge of how the destination country labour market functions, access to social network resources (social capital) (Damm, 2014; Drever & Onno, 2008; Wilson & Alejandro, 1980) as well as strong motivation and readiness to take a risk (Chiswick, 1978; Cohen & Haberfeld, 2007) play positive roles promoting employment chances. Research has also shown that economic incentives may increase the exit rate from public unemployment benefits to employment (Ahmad & Svarer, 2009; Van den Berg, Van der Klaauw, & Van Ours, 2004)‐although recent research seems to indicate that such increase is only a short term effect (Andersen, Dustmann, & Landersø, 2019). Figure 1 provides a logic model that shows the connections between the eight types of programmes and outcomes. It should be emphasised that almost no causal evidence exists for these mechanism. The empirical elements in the existing studies are mainly descriptive.

There may also be unintended effects of some of these interventions. For example, Andersen et al. (2019) shows that use of economic incentives for newly arrived immigrants caused a high increase in property crimes. Furthermore, this study showed that children's likelihood of being enroled in childcare or preschool, their performance in language tests, and their years of education all decreased. We will report on unintended effects if such effects are included in the studies. However, we will not describe the mechanism behind these unintended effects or include these effect in the meta‐analysis. Interventions may also target the environment of the immigrants—for example, employers and community and the matching process between immigrant jobseekers and employers. However, this review will focus on programmes that primarily seek to enhance the employment chances of immigrants through strengthening the resources, level of information and the motivation of the individual immigrant.

### 1.4 Why it is important to do this review

Labour market integration of non‐Western immigrants is a high political priority in many countries that have received non‐Western immigrants and refugees. The OECD has stated that swift access to the labour market affects many other dimensions of refugees' social integration (OECD, 2018a, p.127). Therefore, it is critical to promote integration policies that maximise refugees' access to employment. Migrant specific programmes and general ALMPs are widely deployed as a means of achieving this goal in relation to non‐Western immigrants (OECD, 2018a). Still, we have little knowledge about the effectiveness of these interventions in terms of raising the level of economic self‐sufficiency among members of this target group. Several papers summarise the effects of migrant‐specific programmes and general ALMPs (Arendt & Marie Louise, 2019; Arendt & Pozzoli, 2013; Arendt, Bolvig, Kolodziejczyk, & Petersen, 2016; Butschek & Walter, 2014; Joona & Lena, 2012; Nekby, 2008; Ott & Montgomery, 2015; Rinne, 2013). So far, only Arendt and Pozzoli (2013), Arendt, Bolvig, et al. (2016)^4^ Butscheck and Walter (2014) and Ott and Montgomery (2015) have conducted systematic reviews in this field.

Arendt and Pozzoli (2013) conducted a systematic review of quantitative studies seeking to identify causal effects of interventions to improve non‐Western immigrant self‐sufficiency. They focused on studies seeking to identify a causal effect through either an experimental design (e.g., a lottery), a quasi‐experimental design (e.g., instrumental variables (IVs) regression) or a nonexperimental design (e.g., regression). They focused on effects from interventions on five different outcomes: (a) transition to education, (b) transition to employment, (c) transition to economic self‐sufficiency, (d) duration of unemployment spell, (e) income. Given that they merely found 19 relevant studies, they sought to summarise the effects through a narrative synthesis. The main finding from their review is that wage subsidies in the private sector have positive employment effects. Although important, this review is published in Danish and therefore not accessible to an international audience. Butschek and Walter (2014) also conducted a systematic review seeking to identify which ALMPs are effective for immigrants. Unlike the review outlined in this protocol, their review included all immigrants‐not just non‐Western immigrants. Butscheck and Walter found 33 relevant empirical studies and conducted a meta‐analysis that condensed 93 estimates from these studies. They focussed on effects from ALMPs on immigrants' probability of or hazard to employment. Similar to Arendt and Pozzoli, they found that only subsidised private sector employment can be recommended as a means to improve immigrant's employment outcomes. Although the study by Butscheck and Walter is an important study in this field, as a systematic review it has weaknesses consisting in a lack of transparency and in providing a very limited number of details relating to the search strategy and the screening of relevant literature. Ott and Montgomery (2015) conducted a systematic Campbell review of studies evaluating effects from interventions seeking to improve that economic self‐sufficiency of resettled refugees. The primary outcome was employment rate or labour force participation rate. Secondary outcomes were percentage of the target group receiving specialised refugee cash assistance or public cash assistance, income, job retention and quality of life. The authors found no studies that met the review's inclusion criteria. The review outlined in this protocol includes studies focusing on a broader target group of (non‐Western) immigrants, and hence should have a better chance of identifying relevant studies. The findings from this review should be able to inform policyand decision‐makers at both state‐ and municipal levels as to which labour market programmes are likely to improve economic self‐sufficiency among non‐Western immigrants. Review findings should also provide indications as to the potential size of effects. Hence this review will help decision‐makers choose between different types of programmes based on knowledge of their expected effects.

## 2 OBJECTIVES

This review systematically collects and synthesizes evidence from evaluations of causal effects of interventions designed to improve employment outcomes for non‐Western immigrants. The review aims to answer the following questions:

1) Do interventions designed to improve the economic self‐sufficiency for non‐Western immigrants affect participants employment, use of cash assistance, income, job retention?
2) Do effects differ depending on programme content or populations served?

## 3 METHODS

### 3.1 Criteria for considering studies for this review

#### 3.1.1 Types of studies

In order to summarise what is known about the causal effects of labour market programmes on economic self‐sufficiency of unemployed immigrants we will include all studies with a well‐defined control group. The study designs eligible for inclusion are:

1. Controlled trials
  - Randomised controlled trials (RCTs)
  - Quasirandomised controlled trial designs. Here participants are allocated by means not expected to influence outcomes, for example, alternate allocation, participant's birth data, case number or alphabetic order.
2. Nonrandomised studies where allocation to the intervention and control group are not controlled by the researcher (e.g., by time differences or policy rules). These studies use statistical tools such as differences‐in‐differences models, propensity score matching, regression discontinuity design (RDD) and IVs design based on survey or register data.

These study designs are credible in terms of identifying causal effects from the interventions we investigate. We will include such studies if the quality of each single study is adequately high. We will not include studies without a control group, for example, longitudinal studies estimating effects via comparing average outcomes before and after the intervention. Such studies provide insufficient controls for selection effects and unobserved heterogeneity.

#### 3.1.2 Types of participants

Eligible participants are:

1. Nonemployed job‐seeking immigrants from non‐Western countries residing legally in a Western country. These may or may not be receiving cash‐benefits, unemployment insurance benefits or other kinds of public benefits related to unemployed persons.
2. Immigrants from non‐Western countries residing legally in a western country, who receive cash‐benefits (or similar benefits), but who are characterised by a such a low level of employability (possibly due to health or destination country language problems) that they are not categorised as active job seekers and do not count as unemployed persons in official statistics. Nonetheless, they remain a target group in relation to labour market programmes that aim at increasing their employability, so they can achieve and maintain a job.

Economically inactive groups will be excluded including children, disabled or sick persons, older persons and home makers. Furthermore, the review will not include illegal immigrants. Immigrants can be refugees, displaced persons, economic migrants and persons moving for other purposes, including family reunification.

Western countries are defined as EU28/EEA plus the United States, Canada, Australia and New Zealand. Non‐western countries are all other countries. Individuals belonging to the population fall between the ages of 18 and 64 at the time of intervention. They may vary demographically including geographic, urban/rural, ethnicity and by gender.

The effect estimates need to be estimated on an immigrant sample. We will exclude studies with insufficient information concerning the type of immigrants in the target group, that is, whether such immigrants can be characterised as non‐Western in accordance with the abovementioned definition. In case a study estimates effects for a mixed group of immigrants (both western and non‐Western) we will only include such a study if a majority of immigrants (of no less than two‐thirds of the target group) can be characterised as non‐Western.

#### 3.1.3 Types of interventions

Eligible interventions include programmes designed to increase the economic self‐sufficiency and reduce unemployment rates of immigrants. First, general ALMPs, that is, general programmes also used for the native population; second, migrant specific programmes, that is, programmes specifically designed for and exclusively targeted at immigrants; third, combination programmes that can include combinations of any of the first seven categories (apart from introduction programmes).

General ALMPs comprise four types of interventions

1. (Labour market) training.
2. Subsidised private sector employment.
3. Subsidised public sector employment.
4. Job search assistance.
  We add a fifth type of labour market intervention that is not in itself an ALMP but a typical additional element to ALMPs and some migrant‐specific programmes such as language training:
5. Sanctions and economic incentives.
  Migrant‐specific programmes encompass two categories:
6. Language training.
7. Introduction programmes.
  Combination programmes encompass one category:
8. Combination programmes.

See the Section 1.2 for details on the eight interventions.

#### 3.1.4 Types of outcome measures

##### 3.1.4.1 Primary outcomes

The objective of the included intervention is to increase the economic self‐sufficiency and reduce unemployment rates of non‐Western immigrants. The primary outcome is employment status:

- Unemployment rate/probability/duration
- Employment rate/probability
- Dependency on unemployment insurance benefits or different types of cash assistance (cash assistance may include both general types targeting the general population and immigrant‐specific types of cash assistance, e.g., where the level of cash‐assistance is dependent upon length of stay in the destination country such as the Danish “Start help”).

##### 3.1.4.2 Secondary outcomes

In addition to the primary outcomes, we will include secondary outcomes that are relevant to the impact the described interventions have on duration of employment and the quality of the obtained job, where job quality is measured by wage‐level. The secondary outcomes we will include are:

- Average hourly wage
- Salary/earnings
- Job retention/duration of employment
- Education (enrolment in and completion of lower and upper secondary education, vocational education and higher education)

Inclusion of secondary outcomes does not permit study eligibility.

###### 3.1.4.2.1 Duration of follow‐up

Card et al. (2018) shows that the duration of follow‐up is important for the estimated effect size. Inspired by Card et al. (2018) we consider the following time points for measures:

1) During the intervention (programme)
2) At cessation of the intervention and up to 1 year after the end of the programmed intervention
3) One to two years after the programmed intervention
4) More than 2 years after the programmed intervention

Nonetheless, if the studies provide viable reasons for an adjusted choice of relevant and meaningful duration intervals for the analysis of outcomes, we will adjust the grouping.

### 3.2 Search methods for identification of studies

#### 3.2.1 Electronic searches

Relevant studies will be identified through searches in electronic databases, governmental and grey literature repositories, hand search in specific targeted journals, citation tracking, contact to international experts and internet search engines. The following international databases will be searched:

- Socindex (EBSCO‐host)
- PsycINFO (EBSCO‐host)
- EconLit (EBSCO‐host)
- ERIC (EBSCO‐host)
- Academic Search Premier (EBSCO‐host)
- Science Citation Index (Web of Science)
- Social Science Citation Index (Web of Science)
- Sociological Abstracts (ProQuest)
- IBSS (ProQuest)

##### 3.2.1.1 Description of the search‐string

The search string is based on the PICOs‐model, but only utilises three aspects: population (P), intervention (I) and study type/methodology (s). We have developed three corresponding search facets. Our pilot searches identified a great number of terms for possible outcomes of both primary and secondary nature. Furthermore, we tend to include many different outcomes in the review. Due to the risk of possibly missing key references, we decided not to develop a search‐facet for the outcomes terms, thus including all possible outcomes at the expense of a higher recall. This is also the reasoning for choosing a wide selection of electronic databases covering many fields of potentially relevant references.

The search string includes searches in title, abstract and subject terms for each facet. The subject terms in the facets will be chosen according to each databases options, while the terms used in title/abstract search will remain the same throughout all the database searches.

The following search string (exemplified with a search from Academic Search) will be implemented on the chosen bibliographic databases and modified according to each databases' thesaurus and controlled subject terms.

- S1–4 covers the study type/methodology
- S5–8 covers the population
- S9–12 covers the intervention
- S13 combines the three facets

###### 3.2.1.1.1 Search terms

**Table:** 

Search	Terms
S13	S4 AND S8 AND S12
S12	S9 OR S10 OR S11
S11	DE (“LABOR market” OR “OCCUPATIONAL training for minorities” OR “EMPLOYABILITY” OR “MINORITIES ‐‐ Vocational guidance” OR “EMPLOYMENT of minorities” OR “EDUCATION of migrant labor” OR “EMIGRATION & immigration ‐‐ Economic aspects” OR “FOREIGN workers ‐‐ Government policy”)
S10	AB (job* OR employ* OR unemploy* OR work* OR educat* OR labor* OR labour* OR training* OR language* OR introduct*) N1 (program* OR counsel* OR guid* OR mentor* OR course* OR finding* OR train* OR search* OR initiative*) OR AB (“lab* market*” OR ALMP*)
S9	TI (job* OR employ* OR unemploy* OR work* OR educat* OR labor* OR labour* OR training* OR languag* OR introduc*) N1 (program* OR counsel* OR guid* OR mentor* OR course* OR finding* OR train* OR search* OR initiative*) OR TI (“lab* market*” OR ALMP*)
S8	S5 OR S6 OR S7
S7	DE (“FOREIGN workers” OR “MIGRANT labor” OR “REFUGEES” OR “POLITICAL refugees”)
S6	AB (immigrant* OR migrant* OR asylum* OR refuge*)
S5	TI (immigrant* OR migrant* OR asylum* OR refuge*)
S4	S1 OR S2 OR S3
S3	TI (effect* OR trial* OR experiment* OR random* OR intervent* OR treatment* OR “control group*” “exogenous variation” OR “difference in difference” OR “within household difference*” OR “Regression discontinuity design*”)
S2	AB (effect* OR trial* OR experiment* OR random* OR intervent* OR treatment* OR “control group*” “exogenous variation” OR “difference in difference” OR”within household difference*” OR “Regression discontinuity design*”)
S1	DE (“Randomized Controlled Trials” OR “Experimental Design” OR “STATISTICAL sampling” OR “Clinical Trials” OR “Effect Size (Statistical)” OR “Measurement” OR “CONTROL groups” OR “CASE‐control method” OR “MATCHED groups”)

A full description of each search string used in the searches of the specific databases will be added to the final review.

###### 3.2.1.1.2 Limitations of the search‐string

We will not implement any language or year restrictions to our search.

#### 3.2.2 Searching other resources

##### 3.2.2.1 Hand‐search

We will conduct a hand search of the following journals, in order to make sure that all relevant articles are found. The hand search will focus on editions published between 2015 and 2020 in order to secure recently unpublished articles which have not yet been indexed in the bibliographic databases. A number of specific journals will be hand‐searched. We will decide upon which journals to hand search based on the identified records from the electronic searches. The following are examples of specific journals which we may decide to hand search: Applied Economics, IZA Journal of Migration, Journal of Ethnic and Migration Studies, Journal of Labor Economics and Labour Economics

###### 3.2.2.1.1 Searches for unpublished literature/grey literature

Most of the resources searched for unpublished literature include multiple types of references. As an example, the resources listed to identify reports from national bibliographical resources also include working papers and dissertations, as well as peer‐reviewed references.

We have divided the resources based on the type of the references we expect to identify. In general, there is a great amount of overlap between the types of references in the chosen resources. The resources are listed once under the category of references we expect to be most prevalent in the resource, even though multiple types of unpublished/published literature might be identified in the resource.

##### 3.2.2.2 Search for dissertations

We will search the following resources for dissertations:

- ProQuest Dissertations & Theses Global (ProQuest)
- EBSCO Open Dissertations (EBSCO‐host)

##### 3.2.2.3 Search for working papers/conference proceedings

We will search the following resources for working papers/conference proceedings:

- Google Scholar—https://scholar.google.com/
- Google searches—https://www.google.com/
- Social Science Research Network—https://www.ssrn.com/index.cfm/en/
- NBER Working Papers—http://www.nber.org/papers.html
- IZA—Institute of the Study of Labor—www.iza.org
- MDRC—the Manpower Demonstration Research Corporation—www.mdrc.org
- SOFI Working Papers—https://www.sofi.su.se/english/research/dissertations-and-publications/sofi-working-papers
- IFAU—https://www.ifau.se/
- FRISCH‐Centre—https://www.frisch.uio.no/english/

##### 3.2.2.4 Search for reports/non‐U.S. literature

We will search the following resources for non‐U.S. literature:

- Danish National Research Database—http://www.forskningsdatabasen.dk/en
- SwePub—Academic publications at Swedish universities—http://swepub.kb.se/
- NORA—Norwegian Open Research Archives—http://nora.openaccess.no/
- CORE—research outputs from international repositories—https://core.ac.uk/

##### 3.2.2.5 Search for systematic reviews

Prior to this protocol, we developed a specific search string to identify other systematic reviews in the databases listed above. This was done simultaneously with the development of the search‐string described above, and the identified relevant reviews are considered in this protocol.

Further resources for identifying grey literature might be added during the search process. A final list of grey literature resources will be included in the appendix of the review.

###### 3.2.2.5.1 Citation tracking

In order to identify both published studies and grey literature we will utilise citation‐tracking/snowballing strategies. Our primary strategy will be to citation‐track existing systematic‐reviews and meta‐analyses. The review team will also check reference lists of included primary studies for new leads. We will citation‐track forwards (by using Google Scholar and Web of Science) and backwards (by screening citations in the most relevant literature).

###### 3.2.2.5.2 Contacting international experts

We will contact international experts to identify unpublished and ongoing studies, and provide them with the inclusion criteria for the review along with the list of included studies, asking for any other published, unpublished or ongoing studies relevant for the review. We will primarily contact corresponding authors of the related reviews mentioned in the section Prior reviews, but extend the contacts to others if we find references to or mentions of ongoing studies in screened publications.

### 3.3 Data collection and analysis

#### 3.3.1 Description of methods used in primary research

RCTs are eligible, but we only expect to find few RCTs. Most of the studies are expected to be nonrandomised studies, for instance duration models, matching, RDD or other statistical models. The studies are required to have a control group for inclusion in the review, and methodological appropriateness will be assessed according to the risk of bias assessment models outlined below. Studies with a critical risk of bias will not be included in the data synthesis.

An example of a study that may be included is Delander et al. (2005) who evaluate a Swedish pilot scheme that combine work‐oriented language training and practical workplace training. The aim of the scheme is to enhance the employability of the project participants, but also to prepare them for available training and further education opportunities. Participants are immigrants that the placement officers had found difficult to place in jobs, labour market programmes or regular education because of insufficient knowledge of Swedish. The control groups consist of unemployed immigrants and a propensity score matching method is used to choose members of the control group. The variables used for matching are age, education, citizenship, unemployment benefits, accumulated number of unemployment spells during a 4‐year‐period and accumulated days registered at the PES during a 4‐year‐period. To estimate the effects of the intervention the authors use duration models and look at the effects on:

- Hazard rates, that is (a) effects on transition rates to a job, to a labour market programme or to regular education, and (b) effects on the transition rates to a job.
- Survival functions, that is effects on the probability at different points in time of follow‐up to remain unemployed.

In the estimation of the hazards rates and the survival probability, the authors control for a number of characteristics, for example, gender, age, education, job search, education and experience in wanted profession and accumulated time in unemployment. Another example of a study that may be included is Heinesen et al. (2013), who estimate the effect of ALMPs on the exit rate to regular employment for non‐Western immigrants in Denmark, who receive social assistance. They use a time‐of‐event duration model and estimate the duration of social assistance spell to regular employment simultaneously with the duration from the beginning of the social assistance until entry into ALMPs. The model takes account of nonrandom selection into the programmes with respect to unobservables and observables. The nonparametrical identification of the effects of participating in labour market programmes are based on assuming a mixed proportional hazard and no anticipations effects. The administrative register data used cover all non‐Western immigrants, who began a social assistance spell in 1997 or 1998, and who were 18–66 years of age when the spell began. The control variables in the models are: years since migration, country of origin, type of residence permit, age, family relations, work experience in Denmark, type of municipality, health indicators, local unemployment rate and calendar year.

#### 3.3.2 Selection of studies

Under the supervision of the review authors, two review team assistants will first independently screen titles and abstracts to exclude studies that are clearly irrelevant. Studies that at least one assistant considers eligible or studies with insufficient information in the title and abstract to judge eligibility, will be retrieved in full text. Two review team assistants will, under the supervision of the review authors, independently screen the full texts. The two assistants will compare the result of their screening and discuss disagreements of eligibility. The review authors will be involved in the decision on eligibility, if the two assistants are doubting the eligibility or disagrees regarding the eligibility. The review authors will resolve any disagreement of eligibility. We will document and present in the appendix exclusion reasons for studies that otherwise might be expected to be eligible.

#### 3.3.3 Data extraction and management

The review authors will pilot the study inclusion criteria (see the Appendix 1 “First and second level screening”). A flow diagram will illustrate the overall search and screening process. We will not apply blinding of the review authors to the authors, institutions, or the journals responsible for the publication of the articles. Two review authors will independently code and extract data from included studies. A coding sheet will be piloted on several studies and revised as necessary (see Appendix 2 about data Exstraction). We intend to resolve disagreements between two review authors by consulting a third review author. In case this does not bring unequivocal clarity we will consult an arbiter from the VIVE—Campbell Centre to help decide if a given paper meets the inclusion criteria. Disagreements resolved by a third reviewer or a third reviewer and an arbiter from the VIVE—Campbell Centre will be reported.

Data and information will be extracted on: Available characteristics of participants, intervention characteristics and control conditions, research design, sample size, risk of bias and potential confounding factors, outcomes, and results. Extracted data will be stored electronically. We will conduct analyses using RevMan5 and Stata software. We will code included studies on variables that relate to (a) the methods of the study, (b) the character of the intervention, (c) the characteristics of the subject sample(s), (d) the outcome variables, (e) and contextual features. The list below describes some of the study level variables that we will code for each of these types of characteristics:

1) **Study methods**: Research design (RCT, natural experiment, etc.), statistical methodology (IVs, difference‐in‐difference, matching, etc.), risk of bias, among others.
2) **Character of the intervention**: Main category of intervention (see Section 1.2 and Figure 1), average duration of programme, scale of programme, among others.
3) **Characteristics of the study sample**: Age, gender, length of residence in the destination country, basis for residence permit, family status, among others.
4) **Outcome variables**: (see below).
5) **Contextual features**: Setting, year and type of publication, and so forth. For meta‐analysis we will transform data if needed and appropriate (for details on such transformation see Section 3.3.5 below). In case data necessary for the metaanalysis are missing, we will contact the authors to seek to obtain the necessary data.

#### 3.3.4 Assessment of risk of bias in included studies

We intend to assess risk of bias in RCT studies using Cochranes' risk of bias tool RoB 2 (Higgins, Eldridge, & Li, 2019; Higgins, Savovic, Page & Sterne, 2019). RoB 2 is structured in a fixed set of five domains of bias focussing on different aspects of trial design, conduct and reporting. The five domains are:

1. Bias arising from the randomisation process
2. Bias due to deviations from intended interventions
3. Bias due to missing outcome data
4. Bias in the measurement of the outcome and
5. Bias in selection of the reported result.

Each assessment using the tool focuses on a specific result from a randomised trial. The overall risk of bias for the result is the least favourable assessment across the domains of bias. We will follow the RoB 2 algorithm suggesting a path to domain‐level and overall risk‐of‐bias judgements. However, we will also take into consideration factors that may lead us to override these suggested judgements and justify such decisions in the review (Higgins, Savovic, et al., 2019). We will focus on “risk of material bias,” that is, “issues that are likely to affect the ability to draw reliable conclusion from the study” (Higgins, Savovic, Page, Elbers, & Sterne, 2019, p. 5).

We will use the variants of the RoB 2 tool specific to clusterrandomized trials and crossover trials if studies to be included in the review deploy such methodologies (Higgins, Eldridge, et al., 2019).^5^

For nonrandomised studies of effects of interventions (NRSIs) we intend to use the ROBINS‐I tool (Sterne, Hernán Miguel, et al., 2016). Bias in relation to NRSI can be defined as “the systematic difference between the study results obtained from an NRSI and a pragmatic randomised trial (both with a very large sample size), addressing the same question and conducted on the same participant group, that had no flaws in its conduct” (Sterne, Hernán Miguel, et al., 2016, p. 2). We will use the latest template for completion (Sterne, Higgins & Elbers, 2016).The ROBINS‐I tool is based on the Cochrane RoB tool for randomised trials that was launched in 2008 and modified in 2011. The ROBINS‐I tool covers seven domains through which risk of bias may affect a nonrandomised study.

1. Bias due to confounding
2. Bias in selection of participants into the study
3. Bias in classification of interventions
4. Bias due to deviations from intended interventions
5. Bias due to missing data
6. Bias in the measurement of the outcome
7. Bias in selection of the reported results

The domains included in Robins‐I cover all types of bias potentially present in a NRSI. The first two domains cover types of bias that can be present prior to the intervention while the third domain covers types of bias relating to the intervention itself. The final four domains relate to bias that may arise after the initiation of the intervention.

##### 3.3.4.1 Judgements

The ROBINS‐I tool share many features with the ROB 2 tool. Both tools focus the analysis on a specific result, both are structured into a fixed set of domains of bias, and both include signalling questions that inform risk‐of‐bias judgements. Moreover, both instruments lead to specific and overall risk‐of‐bias judgements that can be overridden with justification.

For randomised studies, answers to the RoB 2 signalling questions lead to judgements of risk of bias in relation to each domain rated on a scale as either “Low”/“Some concerns”/“High”. For NRSI, the answers to the ROBINS‐I signalling questions lead to domain specific judgements rated on a scale as either “Low/Moderate/Serious/Critical/No information”. A “critical” risk of bias implies that a study is too problematic in a particular domain to provide any useful evidence on the effects of the intervention.

We will add the category “critical” to the scale for judging risk of bias in relation to randomised studies in order to obtain equivalent risk‐of‐bias scales for evaluating RCTs and NRSIs. The category “critical” will assume the samemeaning in relation to RCTs as it has in relation to NRSIs. In both cases, a critical risk of bias judgement in a domain implies that we will exclude the related outcome from our data synthesis.

In relation to the RoB 2 assessment, a “high” risk of bias in multiple domains may entail an overall judgement that a “critical” risk of bias affects a particular outcome. The same holds for NRSIs affected by a “serious” risk of bias in multiple domains. In both cases, the implication may be the exclusion of the related outcome from our data synthesis.

##### 3.3.4.2 Confounding

There is a need to investigate how studies deal with confounding factors. Confounding implies that systematic baseline difference between groups may compromise comparability between treatment and control group. Moreover, the ROBINS‐I tool requires review authors to specify important confounding domains and cointerventions in their protocol. Baseline difference in relation to immigrant likelihood of obtaining employment can be observable (e.g., gender, age, educational level), unobservable (e.g., ability, motivation) or difficult to observe (e.g., health or access to aid from social networks). There can be great differences in how studies deal with problems stemming from confounding. Different econometric designs represent different approaches to solving the problem of identifying causal effects from interventions.

Difficulties in estimating causal effects from labour market programmes to improve immigrants' employment outcomes arise from the potential endogeneity that stem from the decision‐making process allocating particular interventions to particular individual immigrants. In some cases, legislation requires that all new immigrants arriving in the destination country after some particular date be treated according to some new intervention—for example, a new training programme, a new social benefits level or some other intervention. In that case a causal analysis of the effects of such an intervention may identify a control group and an intervention group as, respectively, the group of immigrants that arrived just before and just after the date where the intervention was introduced. For example, this is the case in analyses exploring employment effects on newly arrived immigrants from the Danish state lowering in 2002 sharply the level of social benefits for newly arrived immigrants (Andersen et al., 2019; Huynh & Schultz‐Nielsen, 2007).

Still, other types of labour marked interventions targeting immigrants are not universal in scope, and these interventions depend largely on policies at the municipal level or on decisions by individual caseworkers. This may be the case in relation to the type and extent of labour market training received by individual immigrants. This may also be the case when a caseworker decides if an individual immigrant obtains permission to take up publicly subsidised public or private sector employment. Decision‐makers at the local level typically dispose of information on individual immigrants that is not available to the researcher, and therefore estimators of causal effects may be susceptible to bias from different sources.

Therefore, we will look for evidence that the authors of primary studies have a convincing identification strategy, and that they provide reasonable justification for their choice of method. We will assess the extent to which authors deal adequately with risk of bias stemming from unobservable confounders. This assessment is to be based on the list of unobservable confounders that we consider important at the outset (see Appendix *Assessment of risk of bias in included studies*).

In addition to the unobservable confounders, we have identified the following observable confounding factors to be most relevant: length of residence in the destination country, age, education, work experience, gender, parenthood, nationality, and type of residency permit (i.e., economic, humanitarian or family reunification permit). In each study, we will assess whether these factors have been considered.

##### 3.3.4.3 Importance of prespecified confounding factors

Below we provide the motivation for focussing on length of residence in the destination country, age, education, work experience, gender, parenthood, nationality and type of residency permit.

Several labour market studies show that immigrants' employment and earnings rise with their length of stay in the destination countries. Research has explained these findings by gradual skill and information acquisition (Chiswick, 1978; Schultz‐Nielsen, 2019). We also know from a several studies that the age of immigrants at the time of arrival in a new destination country matters to the likelihood of successful labour market integration. Typically, younger immigrants integrate more easily into the labour market than older immigrants (Kogan, 2011). In some countries, the assumption that age (youth) matters to successful labour market integration is reflected in “green card”‐systems, that is, immigration permit point systems, that will only award points relevant to obtaining a residence and work permit to applicants below a certain age (Hawthorne, 2008). Still, the effects from age at migration can be difficult to disentangle from other time related confounders such as age at the onset of an intervention and length of residence in the destination country (Stevens & Ishizawa, 2013). We will primarily look for age at the time of migration and age at the onset of an intervention as potential confounders.

Education and work experience also matter to immigrants' employment success in the destination country (Kogan et al., 2011), although it is important to distinguish education and work experience acquired in the country of origin and education and work experience acquired in the destination country. Education and work experience acquired in the destination country tends to have much larger positive employment effects than education and work experience from the country of origin. Research shows local employers tend to discount qualifications from non‐OECD countries and dismiss foreign work experience almost completely (Damas de Matos & Liebig, 2014). There seems to be limited skill transferability in terms of positive employment effects in the destination country from those qualifications immigrants may have acquired prior tomigration (Arendt & Pozzoli, 2013). Nonetheless, controlling for education and work experience and, if possible, the origin of these qualifications‐whether they were acquired in the country of origin or the destination country‐is important.

Research has also shown that gender and parenthood (Liebig, 2007; Worbs & Baraulina, 2017) matter to employment outcomes of immigrants. Female immigrants participate to a lesser extent in the labour force than male immigrants do, and when they do, their unemployment rate is higher than the equivalent rate of men (OECD, 2018a, p. 81, 107). Moreover, parenthood implies childcare duties that typically affect participation and employment rates of female immigrants negatively to a higher extent than the equivalent rates of male immigrants. Therefore, gender and parenthood are important confounders.

Controlling for type of residency permit is also important, since both participation and employment rates of economic and humanitarian migrants (i.e., refugees) differ. Participation rates of refugees are typically very low in the early period of their stay in the destination country (OECD, 2018a, p. 127; Schultz‐Nielsen, 2019). Likewise, the employment rates of refugees are also lower than those of other types of migrants (Dumont et al., 2016). This is particularly the case of refugee women that is one of the most vulnerable migrant groups (Liebig & Rose, 2018). Participation and employment rates of an accompanying spouse to either an economic migrant or a refugee are typically also lower than the employment rates of natives. Therefore, we will control for type of residency permit distinguishing between economic migrants, humanitarian migrants and family reunification migrants.

The final confounder that we will take into consideration is nationality or region of origin. As stated by the OECD, some migrant groups are facing persistent difficulties in the labour market. This is the case notably for migrants originating in the Middle East and North Africa in Europe and Australia (OECD, 2018a, pp. 82–85). Such difference may arise due to different quality of education across countries or due to discriminatory practices of employers against the hiring of immigrants from particular regions or countries. Irrespective of the underlying cause, we regard region or origin and/or nationality as a potential confounder that we will control for.

##### 3.3.4.4 Effect of primary interest and important cointerventions

We are primarily interested in the effect of being assigned to an intervention at baseline, regardsless of the extent to which the intervention was received during the follow‐up, the so‐called intention‐to‐treat effect (the ITT‐effect). The risk of bias assessments will therefore be in relation to this specific effect. The risk of bias assessments will also consider differences in additional interventions (“co‐interventions”) between intervention groups. Important cointerventions could be health screening programmes and health interventions to help refugees cope with different types of mental or physical health problems (e.g., psychological counselling to traumatised refugees).

##### 3.3.4.5 Assessment

At least two review authors will independently assess the risk of bias for each relevant outcome from the included studies. Any disagreement will be resolved by a third reviewer with content and statistical expertise and will be reported. We will report the risk of bias assessment in risk of bias tables for each included study outcome in the completed review.

#### 3.3.5 Measures of treatment effect

As mentioned earlier the primary outcomes relate to employment. Secondary outcomes relate to earnings and job retention. The primary study outcomes that we will extract fromthe selected studies include unemployment rate, employment probability and hours worked, among others. The secondary study outcomes related to earnings and job retention include average hourly wage, salary and employment duration.

For continuous outcomes we will report mean differences or standardised mean differences (SMD). For outcomes reported on different scales we plan to use the Hedges' g to report SMD. If means and standard deviations is not available, we calculate the SMD's from *F* statistics, *t* statistics, *χ* ^2^ values, and so forth (see Lipsey & Wilson, 2001). If included studies contain too sparse information to conduct these calculations, we will request this information from the principal investigator. For dichotomous outcomes we will report odds‐ratios. We will use 95% confidence intervals.

Salary will probably be an example of a continuous outcome but may also be a dichotomous variable (for example higher or lower than mean‐salary). Employment will probably be a dichotomous variable describing whether the individual has been employed or not, but may also be a continuous variable describing the number of working hours during the year.

There are statistical approaches available the can re‐express oddsratios as SMDs and vice versa, allowing dichotomous and continuous data to be combined in a meta‐analysis (Sánchez‐Meca, Marín‐Martínez, & Chacón‐Moscoso, 2003). We will use this kind of approach where appropriate, that is where an outcome (e.g., salary) can be measured with both binary and continous outcomes.

The outcomes may also bemeasured as durations; for example, the time as unemployed until employment. In such a case the effect will bemeasured as a hazard ratio, where the hazard ratiomeasures the proportional change in hazard rates between unemployed individuals, who are participating in the intervention, and unemployed individuals, who are not participating in the intervention. The hazard rate measures the rate of transition into employment at time *t* conditional on survival as unemployed until time *t*.

The results are probably measured at different time points. As a general guideline, these will be grouped together as follows: (a) during the intervention (programme), (b) at cessation of the intervention and up to 1 year after the end of the programmed intervention, (c) 1–2 years after the programmed intervention and (d) more than 2 year after the programmed intervention. Nonetheless, if the studies provide viable reasons for an adjusted choice of relevant and meaningful duration intervals for the analysis of outcomes, we will adjust the grouping.

#### 3.3.6 Unit of analysis issues

We will take into account whether individuals were randomised in groups, whether individuals have undergone multiple interventions, whether studies use the same sample of data and whether studies use multiple time points.

Cluster randomised trials: we expect that studies typically allocate to the intervention group at the individual level. However, in the case of clustering, for example, at the community level or at the municipal level, we expect that investigators have already controlled for a clustering effect in their results. In cases where authors have not applied methods that control for clustering effects, we will estimate the intra‐cluster correlation (Donner, Piaggio, & Villar, 2001; Hedges, 2007) and correct standard errors.

Multiple intervention groups and multiple interventions per individual: studies with multiple intervention groups with different individuals will be included in the review. Nevertheless, we will only use intervention and control groups that meet the eligibility criteria in the data synthesis. In order to avoid problems with dependence between effect sizes we will apply robust standard errors (Hedges, Elizabeth, & Johnson Matthew, 2010) and use the small sample adjustment to the estimator itself (Tipton, 2015). We will use the results in Tanner‐Smith and Tipton (2014) to evaluate if there are enough studies for this method to estimate the standard errors. In case there are not enough studies, we will use a synthetic effect size (the average) in order to avoid dependence between effect sizes. This method provides an unbiased estimate of the mean effect size parameter but overestimates the standard error. Random effects models applied when synthetic effect sizes are involved actually perform better in terms of standard errors than do fixed effects models (Hedges, 2007). However, tests of heterogeneity when synthetic effect sizes are included are rejected less often than nominal. If pooling is not appropriate (e.g., the multiple interventions and/or control groups include the same individuals), only one intervention group will be coded and compared to the control group to avoid overlapping samples. The choice of which estimate to include will be based on our risk of bias assessment. We will choose the estimate that we judge to have the least risk of bias (primarily, selection bias and in case of equal scoring the incomplete data item will be used).

Multiple studies using the same sample of data: in some cases, several studies may have used the same sample of data or some studies may have used only a subset of a sample used in another study. We will review all such studies, but in the meta‐analysis we will only include one estimate of the effect from each sample of data. This will be done to avoid dependencies between the “observations” (i.e., the estimates of the effect) in the meta‐analysis. The choice of which estimate to include will be based on our risk of bias assessment of the studies. We will choose the estimate from the study that we judge to have the least risk of bias (primarily, selection bias). If two (or more) studies are judged to have the same risk of bias and one of the studies (or more) uses a subset of a sample used in another study (or studies) we will include the study using the full set of participants.

#### 3.3.7 Dealing with missing data

Missing data and attrition rates in the individual studies will be assessed using the risk of bias tool. Studies must permit calculation of a numeric effect size for the outcomes to be eligible for inclusion in the meta‐analysis. Where studies have missing summary data, such as missing standard deviations, we will derive these where possible from e.g., F ratios, *t* values, *χ* ^2^ values and correlation coefficients using the methods suggested by Lipsey and Wilson (2001). If these statistics are also missing, the review authors will contact the principal investigator of the primary study and ask for information on these statistics.

If missing summary data necessary for the calculation of effect sizes cannot be derived or retrieved, the study results will be reported in as much detail as possible, that is, the study will be included in the review but excluded from the meta‐analysis.

#### 3.3.8 Assessment of heterogeneity

The interventions differ with respect to substance and deal with diverse populations (from various countries that differ with respect to constraints in the labour market for immigrants, regulations, how the employment services are organised etc.). We, therefore, expect statistical heterogeneity between primary study outcomes and will use a random effects model in the meta‐analysis, if the number of included studies are sufficient. We will conduct an assessment of heterogeneity using *Q* statistics and its *p* value, the *I* ^2^ statistic, *τ* ^2^ and by visual inspection of forest plots (Borenstein, Higgins, Hedges, & Rothstein, 2017; Higgins & Altman, 2003).

#### 3.3.9 Assessment of reporting biases

If we find a sufficient number of studies, we will use funnel plots to check for possible publication bias (Page, Higgins, & Sterne, 2019). If asymmetry is present, we will consider possible reasons for this. In this context, we are aware that asymmetric funnel plots are not necessarily caused by publication bias and that publication bias not necessarily cause asymmetry.

#### 3.3.10 Data synthesis

When the coding process has been completed, the data will be imported to RevMan5 or STATA to conduct the statistical analysis. We will follow standard procedures for conducting systematic reviews using meta‐analysis techniques, if sufficient studies are identified.

In the meta‐analysis we will exclude studies of low quality. Studies coded with a critical risk of bias will thus be excluded. We expect some relevant studies are using the same sample of data, but in the meta‐analysis we will only use one effect estimate from each sample to avoid dependencies between observations. The choice of estimate to include in the meta‐analysis will be based on the quality assessment of the studies.

We will begin with a descriptive analysis of all the studies. The aim is to present a picture of the existing literature on interventions designed to improve the economic self‐sufficiency and reduce unemployment for non‐Western immigrants. The descriptive analysis will be shown in tables and will report the distribution of the sample of studies with respect to characteristics of the interventions and the participants in the intervention (intervention type, timing and gender of the participants etc.) and the study characteristics (methodology, outcome measure, etc.). Also outcome measures of unintended effects will be reported. Note that we do not plan to include qualitative research in the review.

If there are sufficient studies a meta‐analysis will be performed. As we expect statistical heterogeneity among primary study outcomes, analyses of the overall effect will be inverse variance weighted using random effects statistical models that incorporate both the sampling variance and between study variance components into the study level weights. Effect sizes will be calculated using 95% confidence intervals and we will provide a forest plot of effect sizes. Heterogeneity among primary outcome studies will be assessed with *χ* ^2^ (Q) test, and the *I* ^2^ statistics (Borenstein, Higgins, Hedges, & Rothstein, 2017; Higgins & Altman, 2003; Higgins, Li, & Deeks, 2019). The intention is to conduct a meta‐analysis for each of the primary outcomes.We will also conduct meta‐analyses for the secondary outcomes, if we—against our expectations—find a sufficient number of studies with our secondary outcomes.

We anticipate that several studies provide results separated by for example age and/or gender. We will include results for all age and gender groups. To take into account the dependence between such multiple effect sizes from the same study, we will apply the robust variance estimation approach (Hedges et al., 2010). Different statistical methods may produce effect sizes that are not comparable. For example, analysis using IVs estimates local average treatment effects (LATE), that typically are not directly comparable with average treatment effect from matching. Treatment effects from a RDD may also be LATEs. We will conduct the analysis separately for the LATEs, but also as a sensitivity check include them in the main analysis, depending on the comparability between the LATEs and the other estimates. Note that we include eight different interventions in this review. However, the immigrants often participate in more than one of these intervention at the same time. One example is a Swedish pilot scheme that combine work‐oriented language training and practical workplace training. This scheme is described and evaluated in Delander et al. (2005). Therefore, we will use two different approaches in the meta‐regressions. In the first approach, we include a dummy that describe whether the individual has participated in one of the eight interventions. In the second approach, we include a dummy for each of the eight interventions.

Some studies have shown that the effect of labour market interventions may differ for women and men with immigrant background (Arendt & Schultz‐Nielsen, 2019). Therefore, we will include a dummy for gender in the meta‐regressions and interact this with the intervention variable(s) if there are a sufficient number of studies. If possible, we will carry out subgroup analyses for men and women.

The follow‐up time may also be important and we expect the effect size varies with follow‐up time. We will—if we have a sufficient number of studies—lump the effects for different follow‐up times into one meta‐analysis and add covariates measuring timing, which we interact with the intervention variable(s). If possible, we will also carry out a subgroup analysis for each of the time‐periods mentioned in Section 3.3.5.

Finally, Schultz‐Nielsen (2019) has shown that labour market assimilation varies with type of residence permit. The least successful in the labour market are refugees and family reunions for refugees. Thus, we will to the extent we have a sufficient number of studies interact type of residence permit with the intervention variable(s) and conduct subgroup analyses for categories of type of residence permit.

Following Card et al. (2018), we will include information on destination country in the meta‐analysis as a control‐variable, and also the duration of the programme if possible. We will also—to the extent that it is possible—control for length of residence in the destination country, education, work experience, gender, parenthood and nationality.

#### 3.3.11 Subgroup analysis and investigation of heterogeneity

Previous studies have shown that gender matter to employment outcomes of immigrants (Jakobsen & Liversage, 2017; Liebig & Rose, 2018; OECD, 2017b). Furthermore, a review, primarily focusing on studies from the Nordic countries, find that active labour‐market and social benefit policies have positive effects on employment among immigrant women. However, they are less effective for women than men, whereas the reverse appears to be the case in the long term for policies aimed at skills enhancement (language and formal education; Arendt & Schultz‐Nielsen, 2019). Thus a gender focus is relevant and we will conduct a subgroup analysis for men and women, if the metaregression‐analyses as expected show different effect of the interventions for men and women.

Also type of residence permits (especially whether the immigrant are refugee/family reunited to a refugee or have another type of residence permit; Schultz‐Nielsen, 2019) and follow‐up periods (Card et al., 2018) seem to be important for effect sizes and we will if possible conduct subgroup analyses for categories of type of residence permits and follow‐up periods.

#### 3.3.12 Sensitivity analysis

We will conduct sensitivity analyses by restricting the meta‐analysis to a subset of all studies included in the original meta‐analysis in order to assess whether the pooled effect sizes are robust across components of risk of bias, research design and statistical models in the primary studies.

##### 3.3.12.1 Treatment of qualitative research

We do not plan to include qualitative research

## CONTRIBUTIONS OF AUTHORS

Content: F. T. and V. J. Systematic review methods: F. T. and N. T. D. Statistical analysis: V. J., N. T. D., and F. T. Information retrieval: B. C. V. N.

## DECLARATIONS OF INTEREST

None

## Footnotes

^1^
Own calculations based on OECD‐data. See http://stats.oecd.org/Index.aspx?DataSetCode=MIG

^2^
There are immgirants from some African and Asian countries who are successful in the labour market, for example, immigrants from Japan and South Africa.

^3^
See also Card et al. (2010); Filges et al. (2015).

^4^
Arendt, Bolvig, et al. (2016) is an up date of Arendt (2013) and find four extra studies analysing three different interventions.

^5^
ROBINS‐I: Risk of Bias in Nonrandomised Studies—of Interventions.

## APPENDIX A

### First and second level screening

First level screening is on the basis of title and abstract. Second level is on the basis of full text.

Reference id. No:

Reviewers initials:

Source:

Year of publication:

Country/country of origin:

Author(s):

The study will be excluded if one or more of the answers to screening question 1–4 are “No”. If the answers to the screening questions 1–4 are “Yes” or “Uncertain” then the full text of the study will be retrieved for second level eligibility. All unanswered questions need to be posed again on the basis of the full text. If not enough information is available, or if the study is unclear, the author of the study will be contacted if possible.

#### First and second level screening questions

##### Screening question 1

Does the study focus on the following types of interventions: (1) General ALMPs—(Labour market) training, (2) General ALMPs—Subsidised private sector employment, (3) General ALMPs—Subsidised public sector employment, (4) General ALMPs—Job search assistance, (5) Sanctions and economic incentives, (6) Language training, (7) Introduction programmes, or (8) Combination programmes?

Yes: include

No: if no, then stop here and exclude

Uncertain: include

##### Screening question 1 guidance

In the present review eligible interventions designed to increase the economic self‐sufficiency and reduce unemployment rates of immigrants are: (Labour market) training, Subsidised private sector employment,Subsidised public sector employment, Job search assistance, Sanctions and economic incentives, Language training, Introduction programmes and Combination programmes.

##### Screening question 2

Are the participants 18–64‐years‐old non‐Western immigrants residing legally in western countries?

Yes: include

No: if no, then stop here and exclude

Uncertain: include

##### Screening question 2 guidance

Within the present review, the population is defined as non‐Western immigrants between 18 and 64 years of age residing legally in Western countries. Immigrants can be refugees, displaced persons, economic migrants and persons moving for other purposes, including family reunification. Western countries are defined as EU28/EEA plus the United States, Canada, Australia and New Zealand. Non‐Western countries are all other countries.Studies may include data from other populations, but in order to be included in the present review, the included primary studies must either report or permit us to extract separate effect sizes for the population of this review.

##### Screening question 3

Does the study examine a labour market outcome, such as: unemployment rate, unemployment probability, unemployment duration, employment rate, employment probability, average hourly wage, salary/earnings, job retention/duration of employment?

Yes: include

No: if no, then stop here and exclude

Uncertain: include

##### Screening question 3 guidance

The present review examines the interventions designed to improve economic self‐sufficiency, and any outcome related to economic self‐sufficiency may be relevant. Therefore the list of potentially relevant outcomes is not exhaustive. For instance, if a study reports number of hours worked during a time period following the intervention, this outcome will also be included. However, studies focusing on postintervention outcomes such as mental health, linguistic ability or knowledge of the destination country will not be included.

##### Screening question 4

Is the report/article a quantitative evaluation study with a comparison condition?

Yes: include

No: if no, then stop here and exclude

Uncertain: include

##### Screening question 4 guidance

We are only interested in primary quantitative studies with a comparison group, where the authors have analysed the data. We are not interested in theoretical papers on the topic or surveyes/reviews of studies of the topic (this question may be difficult to answer based on title and abstracts).

### DATA EXTRACTION

**Table:**

Names of author(s)
Title
Language
Journal
Year
Country
Setting (interventions assigned by public, that is, state, regional or municipal, authorities, or interventions assigned by private for‐profit or nonprofit actors on behalf of public authorities)
Type of publication
Characteristics of the intervention (type of intervention, duration of programme, scale of programme)
Study methods (Research design, Statistical methodologies)
Characteristics of the study sample (age, gender, length of residence in the destination country, basis for residence permit, family status)
Outcome variables (unemployment rate, unemployment probability, unemployment duration, employment rate, employment probability, average hourly wage, salary/earnings, job retention/duration of employment)
Type of data used in the study (administrative register data, questionnaire, other (specify))
Sample size (divided into treated/comparison)

#### Outcomes

Instructions: Please enter outcome measures in the order in which they are described in the report. Note that a single outcome measure can be completed by multiple sources and at multiple points in tome (data from specific sources and time‐points will be entered later)

**Table:**

#	Outcome & measure	Reliability & validity	Format	Direction	Source	Pg# Notes
1		Info from:	Dichotomy	High score or event is	Questionnaire	
		Other samples	Continuous	Positive	Admin. register data	
		This sample	Time‐to‐event	Negative	Other (specify)	
		Unclear		Can't tell	Unclear	
		Info provided				

Repeat as needed

OUTCOME DATA: DICHOTOMOUS OUTCOME DATA

**Table:**

	Time points						
Outcome	(record exact time from participation, there may be more than one, record them all)	Source	Valid Ns	Cases	Non‐cases	Statistics	Pg# Notes
		Questionnaire	Participation	Participation	Participation	RR (risk ratio)	
		Admin. register data				OR (Odds ratio)	
		Other (specify)	Comparison	Comparison	Comparison	SE (standard error)	
		Unclear				95% CI	
						DF	
						Exact p‐value	
						Chi2	
						Other	

Repeat as needed

OUTCOME DATA: TIME‐TO‐EVENT OUTCOME DATA

**Table:**

	Time points				
Outcome	(record exact time from participation, there may be more than one, record them all)	Source	Methods of estimation	Statistics	Pg# Notes
		Questionnaire	Non‐parametric	HR(hazard ratio)	
		Admin. register data	Semi‐parametric	SE (standard error)	
		Other (specify)	Parametric	95% CI	
		Unclear		DF	
				Exact p‐value	
				Chi2	
				Other	

Repeat as needed

OUTCOME DATA: CONTINUOUS OUTCOME DATA

**Table:**

	Time points						
Outcome	(record exact time from participation, there may be more than one, record them all)	Source	Valid Ns	Means	SDs	Statistics	Pg# Notes
		Questionnaire	Participation	Participation	Participation	P	
		Admin. register data				t	
		Other (specify)	Comparison	Comparison	Comparison	F	
		Unclear				Df	
						ES	
						Other	

Repeat as needed

### Assessment of risk of bias in included studies

#### User guide for unobservables

Systematic baseline differences between groups can compromise comparability between groups. Baseline differences can be observable (e.g., age and gender) and unobservable (to the researcher; e.g., motivation and “ability”). There is no single nonrandomised study design that always solves the selection problem. Different designs solve the selection problem under different assumptions and require different types of data. How different designs deal with selection on unobservables varies. The “right” method depends on the model generating participation, that is, assumptions about the nature of the process by which participants are selected into a programme.

Since there is no universally correct way to construct counterfactuals, we will assess the extent to which the identifying assumptions (the assumption that makes it possible to identify the counterfactual) are explained and discussed. Preferably the authors should make an effort to justify their choice of method. We will look for evidence that authors using one of the following methods (this is not an exhaustive list) discuss key identifying assumptions in relation to the method chosen. More specifically we will look for whether they:

##### Natural experiments

Discuss whether the natural experiment provides a truly random allocation of participants and that there is no change of behaviour in anticipation of, for example, policy rules.

##### Matching (including propensity scores)

Explain and discuss the assumption that there is no selection on unobservables, only selection on observables.

##### (Multivariate, multiple) regression

Explain and discuss the assumption that there is no selection on unobservables, only selection on observables. Further discuss the extent to which they compare comparable people.

##### Regression discontinuity (RD)

Explain and discuss the assumption that there is a (strict!) RD treatment rule. It must not be changeable by the agent in an effort to obtain or avoid treatment. Continuity in the expected impact at the discontinuity is required.

##### Difference‐in‐difference (treatment‐control‐before‐after)

Explain and discuss the assumption that the trends in treatment and control groups would have been parallel, had the treatment not occurred.
